# Factors that affect the assessment of the quality of life of rheumatoid arthritis patients depending on the prevalence of frailty syndrome

**DOI:** 10.1186/s12955-020-01472-3

**Published:** 2020-07-06

**Authors:** E. Bąk, A. Młynarska, C. Marcisz, R. Bobiński, D. Sternal, R. Młynarski

**Affiliations:** 1 Faculty of Health Sciences, University of Bielsko-Biala, ul. Willowa 2, 43-309 Bielsko-Biala, Poland; 2 Department of Gerontology and Geriatric Nursing, School of Health Sciences, Medical University of Silesia, Katowice, Poland; 3 Department of Electrocardiology and Heart Failure, School of Health Sciences, Medical University of Silesia, Katowice, Poland

**Keywords:** Frailty, Rheumatoid arthritis, Quality of life

## Abstract

**Abstract:**

Rheumatoid arthritis (RA) has a large and varied impact on the quality of life as associated with patient health including both physical and mental well-being.

The aim of the study was to assess the factors that affect the assessment of the quality of life of RA patients depending on the prevalence of frailty syndrome.

**Material and methods:**

The study involved 106 patients with RA (82 women; mean age 65.83 ± 5.01), who had been hospitalized in the Silesian Centre for Rheumatology, Rehabilitation and Disability Prevention in Ustron, Poland. The patients that were included in the study were divided into two groups depending on the incidence of frailty syndrome: Group 1 – robust patients and Group 2 – patients with frailty syndrome.

**Results:**

Frailty syndrome was identified in 34.9% of the patients with recognized/diagnosed RA; in women, it was 36.14% and in men, it was 25.92%. The average TFI value was 4.11 ± 2.05; in the physical domain, it was 3.39 ± 1.66; in the mental domain, it was 0.41 ± 0.55 and in the social domain, it was 0.31 ± 0.48. The robust patients assessed their quality of life associated with sleep as being worse compared to patients with recognized frailty syndrome.

**Conclusion:**

Frailty syndrome has no significant impact on the assessment of the quality of life of patients with diagnosed RA. The factors that determine quality of life are different in robust patients and in patients with frailty syndrome. The assessment of the quality of life is affected by the degree of an individual’s fitness regardless of the occurrence of frailty syndrome.

## Introduction

Rheumatoid arthritis (RA) is the most common chronic inflammatory disease of the joints. RA affects about 1% of the population around the world. The disease can start at any age, but the peak of the illness is observed between 30 and 50 years of age [1, 2]. Women suffer three times more often than men. In about 30% of cases, RA only occurs after 60 years of age – this is very important because as life expectancy is extended, the number of older people with newly diagnosed RA will also increase [3]. In this illness, disability is both common and significant. In a large cohort in the United States, 35% of RA patients were not able to work after 10 years [4]. An older age, a positive family history of RA and the female gender are associated with an increased risk of RA, although the gender differences are less pronounced in older patients [1].

Rheumatoid arthritis has a significant and varied impact on the quality of life as associated with patient health (Health Related Quality of Life – HRQoL) including both physical and mental well-being [5]. Measuring quality of life as conditioned by the state of health is based on a subjective assessment of patients in the physical, mental and social aspects. It provides some insight into the severity of any symptoms and side effects that affect a patient’s quality of life. People with RA often report a decrease in their HRQoL, which can be characterized as the impact of the disease on their physical, emotional and social health. People with RA have a HRQoL that is not as good as people with other rheumatic diseases or their healthy contemporaries. A lower assessment of quality of life may even persist when the disease is well controlled. The quality of life of RA patients is affected by fatigue, pain, stiffness and impaired physical functioning [6, 7]. Some socio-economic factors such as age, employment, economic status and lifestyle habits also affect their quality of life [8].

Explaining frailty etiology and its natural history is therefore critical for identifying high risk subpopulations and new areas for the prevention and treatment of frailty [9–13]. Frailty is theoretically defined as a clinically recognizable state of increased vulnerability that results from an aging-associated decline in reserve and function across multiple physiologic systems such that the ability to cope with every day or acute stressors is reduced [14–16]. Frailty is a common clinical syndrome in older adults that carries an increased risk for poor health outcomes including falls, incident disability, hospitalization and mortality [17].

The aim of the study was to assess the factors that affect the assessment of the quality of life of RA patients depending on the prevalence of frailty syndrome.

## Methods

The study was performed in the Silesian Centre for Rheumatology, Rehabilitation and Disability Prevention in Ustron, Poland. Based on the size of the population (1% of the adult population), fraction size and maximum error (2%) at the 95% confidence level, the minimum number of people in a sample of 96 persons was calculated. The data that were used to calculate the minimum number of individuals in the group were obtained from a panel of experts from the National Consultant on Rheumatology [18]. A physical examination, anthropometric examination and laboratory tests were additionally performed in all of the individuals that were included in the study. The patients that were included in the study were divided into two groups depending on the incidence of frailty syndrome:

- Group 1 – robust patients
- Group 2 – patients with a frailty syndrome

### Inclusion criteria

- A diagnosis of RA based on the ACR/EULAR 2010 diagnostic standards,
- age ≥ 60 years,
- consent to participate in the study

### Exclusion criteria

- diagnosed cancer in the active phase,
- previously diagnosed mental illness or stroke,
- incomplete questionnaire

Participation in the study was anonymous and voluntary. The consent of the Bioethics Committee of the Beskidzka Regional Chamber of Physicians in Bielsko-Biala (No. of consent 2017/02/16/6) was obtained for the trial. The study was conducted in accordance with the guidelines of the Helsinki Declaration and the principles of Good Clinical Practice that were in effect at the time of the study.

### Instruments used in the research

#### Nottingham Health Profile – NHP

The NHP is a QoL clinimetric index questionnaire that has been used since 1986. The questionnaire is divided into two parts, the first of which consists of 38 questions concerning the main areas of life such as pain, energy, sleep, emotional reactions, physical mobility and social isolation. The second part consists of seven questions regarding housework, employment, social and sexual life, personal relationships, how holidays and free days are spent and hobbies and interests [19]. The higher the score, the more severe the health problem (up to 100 points can be obtained in both parts). The Polish adaptation of the NHP was prepared with the consent of the authors of the questionnaire.

#### Tilburg Frailty Indicator – TFI

All of the patients were assessed to determine the development of frailty syndrome using the Tilburg Frailty Indicator scale. The TFI consists of two parts: the determinants of frailty syndrome (age, sex, marital status, level of education and lifestyle) and the components of frailty. The components of the frailty consist of 15 questions that are ranked according to three different domains: the physical domain, the psychological domain and the social domain. The values of the indicators can range from 0 to 15 and frailty syndrome is recognized as a score of at least five points. The TFI is characterized by its high ability to detect multidimensional deficits, which makes it an appropriate method for testing frailty syndrome for preventive purposes [20]. This tool was translated into Polish and its clinical value has been confirmed in numerous studies [21, 22].

#### Health Assessment Questionnaire – HAQ

The Health Assessment Questionnaire (HAQ) enables the long-term effects of a chronic illness on a patient’s life to be assessed. The HAQ was created to enable the degree of improvement that is obtained at subsequent stages of treatment in patients with rheumatic diseases to be compared. This research tool includes 20 questions in eight categories. The questions relate to the functional sphere: hygiene, dressing, standing up from various body positions, lifting items, eating, moving and grasping. The HAQ score ranges from 0 to 3 and can be calculated when the patient has completed at least three sections [23].

#### Disease Activity Score – DAS28-CRP

The disease activity was assessed using the Disease Activity Score, which is calculated from 28 joints (DAS 28) [24]. The indicators of inflammation are measured using the standard laboratory parameters and the concentration of the C-reactive protein (CRP, C-reactive protein).

### Charlson Comorbidity Index (CCI)

The method for classifying comorbidity created by Charlson et al. is a simple and valid method for estimating the risk of death from comorbid diseases that is used in longitudinal studies [25].

### Statistical analysis

The data was analyzed using Statistica 13.1 software. A study of the normality of the quantitative variables was performed using the Kolmogorov-Smirnov test. For those variables that did not show a normal distribution, a significance test was performed using the non-parametric Mann-Whitney U-test. Conversely, for variables with a normal distribution, the level of significance of the differences between frailty syndrome was determined using the Student’s t-test. Difference significance tests for the qualitative data were performed using the Chi^2^ test. In order to assess whether the analyzed parameters were predictors of the dependent variables, multiple regression analysis using the stepwise method was used. A significance level of 0.05 was assumed in the calculations.

## Results

The study involved 106 patients with RA (82 women; mean age 65.83 ± 5.01), who had been hospitalized in the Silesian Centre for Rheumatology, Rehabilitation and Disability Prevention in Ustron, Poland. Frailty syndrome was identified in 34.9% of the patients with recognized/diagnosed RA; in women, it was 36.14% and in men, it was 25.92%. The average TFI value was 4.11 ± 2.05; in the physical domain, it was 3.39 ± 1.66; in the mental domain, it was 0.41 ± 0.55 and in the social domain, it was 0.31 ± 0.48. The average Charlson Comorbidity Index value in the study group was 2.07 ± 0.99; there was no statistically significant difference between the robust patients and those with diagnosed frailty syndrome (1.98 ± 1.05 vs 2.22 ± 0.85; *p* = 0.2538). The characteristics of the patients that were included are presented in Table 1.

**Table 1:** Characteristics of the patients that were included in the study

**Parameters**	**Whole group**			**Robust (69)**			**Frail (38)**			**p**
Place of living (urban/rural area)	29/77			16/53			13/24			0.79
Education (primary/vocational/ secondary/higher)	15/34/37/20			9/20/28/12			6/14/9/8			0.95
Marital Status (unmarried/married/ living with partner/widow;widower or divorced)	2/69/29/6			1/45/19/4			1/24/10/2			1
Professional status (working/ not working/retired/pensioner on benefits)	10/10/17/69			4/4/13/48			6/6/4/21			0.52
Type of work (physical/mental)	59/47			37/32			22/15			0.99
Drinking coffee (n): yes/no	89			59			30			0.99
Drinking alcohol (n): yes/no	20			13			7			1
Drugs (n): Methotrexat / NSAiD/ glucocorticosteroids/ etanercept/ Adalimumab	106/20/24/18/12			69/17/20/12/7			37/15/11/6/5			0.74
	**median**	**1st quartile**	**3rd quartile**	**median**	**1st quartile**	**3rd quartile**	**median**	**1st quartile**	**3rd quartile**	**p**
Age (years)	65	62	68	65	63	68	65	62	68	0.75^a^
Duration of disease (months)	142	72	240	180	90	240	120	60	240	0.20
Number of painful joints (n)	6	4	8	6	4	10	6	4	8	0.49
Number of swollen joints (n)	4	2	6	4	2	7	4	2	4	0.24
Duration of morning stiffness (minutes)	60	30	60	60	30	60	45	20	60	0.22
BMI (kg/m^2^)	26.89	24.09	29.35	26.93	23.8	29.35	26.84	24.8	29.24	0.46
VAS	55	45	65	55	45	65	56	42	65	0.68^a^
Red blood cell	4.34	4.12	4.68	4.32	4.12	4.6	4.41	4.14	4.8	0.09
ESR	17.5	13	28	20	13	32	16	13	20	0.05
CRP	0.73	0.42	1.48	0.74	0.49	1.52	0.7	0.42	1.25	0.38
Creatine	0.76	0.7	0.85	0.78	0.7	0.85	0.73	0.7	0.84	0.11
ALAT	22	17	28	21	16	24	23	19	31	0.01^a^
ASPAT	22	18	25	22	18	24	22	18	25	0.44^a^
DAS-28	4.71	4.24	5.38	4.86	4.16	5.64	4.6	4.26	4.95	0.02^a^
DAS-28-CRP	3.92	3.41	4.58	3.99	3.41	4.69	3.84	3.41	4.20	0.049^a^
Acceptance of illness	27	21	33	26	20	32.5	30	23	34	0.28^a^
Smoking (nonsmoker/smoker) Number of days	15			8			7			0.90
	10	5	15	10	6	12.5	10	5	18	0.97^a^
Smoking in the past Number of days Number smoking years	35			25			10			0.92
	10	5	20	10	5	20	10	7	20	0.51^a^
	20	15	30	20	15	30	22	15	30	0.39^a^

*Abbreviations*: *AlAT* alanine transaminase, *AspAT* aspartate transaminase, *BMI* body mass index, *CRP* C-reactive protein, *DAS-28* 28-Joint Disease Activity Score, *ESR* erythrocyte sedimentation rate, *IQR* interquartile range, *P* statistical significance of differences, *VAS* Visual Analog Scale

^a^T-Student test

The quality of life analysis showed no significant differences between patients diagnosed with frailty syndrome compared to robust in all domains except the domain with sleep problems. Robust patients assessed the quality of life associated with sleep as being worse compared to patients with recognized frailty syndrome. The details of the quality of life assessment for the entire group and those that were dependent on the prevalence of frailty syndrome are presented in Table 2.

**Table 2:** Quality of life assessment for the whole group and the assessment depending on the prevalence of frailty syndrome

Analyzed parameters	Whole group			Robust (69)			Frail (38)			p
	median	1st quartile	3rd quartile	median	1st quartile	3rd quartile	median	1st quartile	3rd quartile	
**NHP-EL**	76	37	100	76	39	100	61	24	100	0.17^b^
**NHP-P**	66	43	87	64	44	85	66	41	89	0.73^a^
**NHP-ER**	86	61	100	86	67	100	78	54	90	0.16^b^
**NHP-S**	78	40	100	87	50	100	50	35	84	0.008^b^
**NHP-SI**	100	78	100	100	78	100	100	80	100	0.79^b^
**NHP-PA**	67	46	87	67	46	78	69	54	87	0.61^a^
**NHP (n [%])**										
**Paid employment**	33 (31.13%)			21 (30.43%)			12 (32.43%)			0.99
**Housework**	64 (60.38%)			38 (55.07%)			26 (70.27%)			0.68
**Social life**	24 (22.64%)			14 (20.29%)			10 (27.03%)			0.96
**Family life**	19 (17.92%)			8 (11.59%)			11 (29.73%)			0.25
**Sex life**	27 (25.47%)			14 (20.29%)			13 (35.14%)			0.59
**Hobbies**	50 (47.17%)			31 (44.93%)			19 (51.35%)			0.98
**Holidays**	49 (46.23%)			32 (46.38%)			17 (45.95%)			1

*Abbreviations*: *EL* energy level, *ER* emotional reaction, *NHP* Nottingham Health Profile, P pain, *P* statistical significance of the differences, *PA* physical abilities, *S* sleep, *SI* social isolation

^a^T-Student test

^b^Mann-Whitney U-test

A multiple regression analysis was performed in order to determine the relationships between the sociodemographic and clinical factors of RA and the level of quality of life. A multiple regression model in which the predictors were the biochemical parameters, sociodemographic variables (years of education, sex, education, marital status, alcohol consumption and BMI) and functioning in everyday life (problems in home life, current state of health, problems doing housework, problems on vacation and the intensity of pain on the VAS scale) and the dependent variable energy level (EL) were statistically significant and explained 99% of the observed variation in the dependent variable (*p* < 0.0001, *R2* = 0.99). The results are presented in Table 3.

**Table 3:** Factors affecting the energy level and pain depending on the prevalence of frailty syndrome

	Energy level				Pain			
	Robust		Frail		Robust		Frail	
	X_i_	p	X_i_	p	X_i_	p	X_i_	p
**Interconcept**	0.14	0.54	0.86	0.001	1.27	0.004	−2.21	< 0.001
**Age**					−0.01	0.007	–	–
**Pension**	0.56	< 0.001	1.26	< 0.001				
**Retired**					−0.16	0.002	–	–
**Professional status - working**					−0.32	0.002	–	–
**Problems with family life**	0.22	0.006	–	–				
**ASPAT**	0.02	0.001	–	–	0.03	< 0.001	–	–
**BMI**	−0.02	0.005	−0.04	< 0.001				
**CRP**	0.01	< 0.001	–	–	–	–	0.46	0.002
**Current health - good**	−0.58	< 0.001	–	–	−0.64	< 0.001	–	–
**HAQ**	0.26	< 0.001	0.24	< 0.001				
**Number of years smoking**	0.01	0.005	–	–				
**Widower/widow**	0.20	0.04	–	–				
**Married**					0.32	< 0.001	–	–
**Problems with housework**	–	–	0.44	< 0.001	–	–	0.17	0.01
**Problems with holidays**	–	–	0.21	0.002				
**Education vocational**	–	–	−0.35	< 0.001				
**Education - secondary**					−0.41	< 0.001	–	–
**Drinking alcohol**	–	–	−0.26	< 0.001				
**Gender**	–	–	0.06	0.02				
**VAS**	–	–	–	–	–	–	0.03	< 0.001
**R**^**2**^	0.94		0.97		0.93		0.97	
**P - Anova**	< 0.0001		< 0.0001		< 0.0001		< 0.0001	

*Abbreviations*: *AspAT* aspartate transaminase, *BMI* body mass index, *CRP* C-reactive protein, *DAS-28* 28-Joint Disease Activity Score, *ESR* erythrocyte sedimentation rate, *HAQ* Health Assessment Questionnaire, *IQR* interquartile range, *P* statistical significance of differences, *VAS* Visual Analog Scale

In the next model of multiple regression, the quality of life – pain (P) was the dependent variable. The results for the entire group and those that were dependent on a recognition of frailty syndrome are presented in Table 3. The level of the perception of pain is affected by a high level of the erythrocyte sedimentation rate (ESR). However, current good health, creatinine level and the HAQ have a negative impact. Moreover, a lower level of pain sensation occurred in people with a secondary education and retirees. People who smoked and people with problems with their free time activities experienced a greater intensity of pain. The R^2^ ratio showed that the obtained model explained 84% of the variation in the perception of the level of pain.

In the other model, the quality of life – emotional reaction (ER) was the dependent variable. The results are presented in Table 4. In the robust group, BDI, civil status and the level of frailty in the physical domain had a positive impact on the emotional reaction level. However, problems with hobbies, housework, education and the place of residence had a negative effect. The coefficient R^2^ showed that the obtained model explained 97% of the variation in the emotional reaction (*R2* = 0.97, *p* < 0.0001). However, in the group with frailty syndrome, only a bad state of health was the factor that affected the assessment of quality of life. The coefficient R^2^ showed that 57% of the variation in the emotional reaction was explained by this model.

**Table 4:** Factors affecting the emotional reaction and sleep depending on the prevalence of frailty syndrome

	Emotional reaction				Sleep			
	Robust		Frail		Robust		Frail	
	X_i_	p	X_i_	p	X_i_	p	X_i_	p
**Interconcept**	0.93	< 0.001	0.74	< 0.001	−1.54	0.003	0.68	0.001
**BDI**	0.02	< 0.001	–	–	–	–	0.01	0.003
**Drinking alcohol**	–	–	–	–	0.83	0.005	–	–
**Gender**	–	–	–	–	0.02	0.03	–	–
**Current health - worse**	–	–	0.67	0.03	–	–	–	–
**Smoking in the past**	–	–	–	–	0.13	0.01	–	–
**DAS28-CRP**	–	–	–	–	0.10	0.004	–	–
**Problems with hobbies**	0.21	< 0.001	–	–	–	–	–	–
**Problems with paid employment**	–	–	–	–	1.78	0.003	0.12	0.03
**Problems with housework**	0.11	< 0.001	–	–				
**Problems with holidays**					–	–	0.57	< 0.001
**Creatine**	–	–	–	–	1.52	0.004	–	–
**Age**	–	–	–	–	0.03	0.003	–	–
**Pension**	–	–	–	–	0.47	0.003	–	–
**Education - vocational**	−0.16	< 0.001	–	–	–	–	–	–
**Married**	0.10	0.002	–	–	–	–	–	–
**Place of living**	−0.05	0.009	–	–				
**Number smoking years**					0.01	0.004	–	–
**Education - secondary**					−1.28	0.001	–	–
**Place of living**					−0.24	0.003	–	–
**Current health - good**					−0.65	0.001	–	–
**Drinking coffee**					0.47	0.003	–	–
**Education - higher**					−0.33	0.009	–	–
**R**^**2**^	0.97		0.57		0.99		0.99	
**P - Anova**	< 0.0001		0.03		0.001		< 0.001	

*Abbreviations*: *AspAT* aspartate transaminase, *BMI* body mass index, *CRP* C-reactive protein, *DAS-28* 28-Joint Disease Activity Score, *ESR* erythrocyte sedimentation rate, *HAQ* Health Assessment Questionnaire, *IQR* interquartile range, *P* statistical significance of differences, *VAS* Visual Analog Scale

Analysis of the multivariate regression model in which the dependent variable was the quality of life in the sleep domain showed that the model was statistically significant and explained 98% of the observed variability in the dependent variable (*p* < 0.0001, *R*^*2*^ = 0.98). In both the robust and frail patients, the model explained 99% of the observed variations in the dependent variable (*p* < 0.001, *R*^*2*^ = 0.99). Detailed data for the entire group as well as for the subgroups is presented in Table 4.

Table 5 present the multivariate regression model for physical mobility and social isolation for patients with and without recognized frailty syndrome. The models that are presented are statistically significant. In both of the presented alternative models, the dependence in the patients with frailty syndrome was determined by variables other than those for the robust patients.

**Table 5:** Factors affecting social isolation and physical abilities depending on the prevalence of frailty

	Social isolation				Physical abilities			
	Robust		Frail		Robust		Frail	
	X_i_	p	X_i_	p	X_i_	p	X_i_	p
**Interconcept**	1.66	< 0.001	0.47	0.001	0.22	0.20	0.32	< 0.001
**BDI**	–	–	–	–	0.02	< 0.001	–	–
**Current health - worse**	0.44	< 0.001	–	–	0.38	0.004	0.02	0.002
**Current health - good**					−0.16	0.02	–	–
**Working**	0.29	< 0.001	–	–				
**Problems with holidays**	−0.15	< 0.001	–	–				
**Problems with family life**	0.18	< 0.001	–	–				
**Problems with hobbies**					0.19	< 0.001	–	–
**Problems with social life**					−0.37	< 0.001	–	–
**CRP**	0.008	< 0.001	–	–				
**Problems with sex life**	0.12	0.001	–	–				
**Divorced**	−0.13	0.001	–	–				
**Education - higher**	0.01	0.004	–	–	−0.41	< 0.001	–	–
**HAQ**	0.02	0.003	–	–	0.10	0.01	0.36	< 0.001
**Retired**	0.05	0.001	–	–				
**Acceptance of disease**	−0.002	0.002	–	–				
**Red blood cell**	−0.0001	0.005	–	–	−0.004	< 0.001	–	–
**BMI**	0.0002	0.02	–	–	–	–	0.02	< 0.001
**Duration of disease**	0.00001	0.03	–	–	–	–	0.001	< 0.001
**Married**	–	–	0.46	0.002				
**DAS28-CRP**					0.18	< 0.001	–	–
**Smoking in the past**					−0.26	< 0.001	–	–
**Drinking alcohol**					−0.24	< 0.001	–	–
**R**^**2**^	0.99		0.78		0.98		0.99	
**P - Anova**	< 0.0001		0.002		< 0.0001		< 0.001	

*Abbreviations*: *AspAT* aspartate transaminase, *BMI* body mass index, *CRP* C-reactive protein, *DAS-28* 28-Joint Disease Activity Score, *ESR* erythrocyte sedimentation rate, *HAQ* Health Assessment Questionnaire, *IQR* interquartile range, *P* statistical significance of differences, *VAS* Visual Analog Scale

## Discussion

Although the impact of frailty syndrome on the mortality of older RA patients is unclear, a few observational studies of patients with osteoarthritis have shown that frailty and its associated geriatric syndromes (GS) increase the risk of long-term mortality in elderly patients [26]. The research of Salaffi et al., which examined the prevalence of frailty in adult patients with rheumatoid arthritis, is important in this subject [27]. The authors used the Survey of Health, Ageing and Retirement in Europe Frailty Instrument (SHARE-FI) tool that was created by Santos-Eggimann et al. [28] They confirmed that frailty or pre-frailty are common in this disease. The advantage of their research is the use of a device for measuring muscle strength. This increased the measurement of frailty objectivity compared to the TFI that we used. However, the results seem to be comparable. There is no complete study in the literature about the impact of frailty syndrome on the quality of life of patients with known RA. Elderly patients with advanced RA are more likely to progress to frailty syndrome because they are more likely to experience a functional decline, depression, cognitive impairment, falls, malnutrition and polyphagia. Treating patients with attendant frailty syndrome is usually difficult and complicated because they often have different coexisting diseases that are related to age, RA and treatment and are prone to progress to an irreversible stage of disability [29, 30].

The DAS28, which evaluates joint tenderness, edema, inflammatory biomarkers and the general health of a patient, is a proxy indicator for the severity of RA and confirmed a relationship with functional capacity [31]. Dunlop et al. reported that in elderly patients with arthritis, disability was associated with an older age, cognitive dysfunction and depressive symptoms, which are important components of an overall geriatric evaluation [32]. A study of 100 patients with RA showed that a longer duration of the disease was positively associated with functional disability, which could result in GS. In this study, we found that older RA patients with a higher level of disease activity (as defined by DAS28), a longer disease duration and an impairment of their physical functions (as determined by a higher HAQ score) were more susceptible to developing GS. However, only a higher DAS28 was an independent risk factor for GS. In our study, we showed a statistically significant difference between patients with diagnosed, but not recognized, frailty syndrome in terms of the DAS28 and DAS28CRP, which may confirm the conclusion that the DAS28 is an independent factor in developing GS and frailty syndrome [33, 34]. Patients with RA have more pain compared to the entire population and have similar levels to patients with normal pain, although their level of disability is higher. It has been shown that higher pain levels correlate with disability and with depression, all of which significantly contribute to the quality of life of RA patients. Clinically significant fatigue occurs in 40–80% of patients with RA [35–37].

The Health Assessment Questionnaire (HAQ), which was also used in our research, assesses the long-term effects of a chronic illness on a patient’s life. The HAQ was created to enable the degree of improvement that is obtained at subsequent stages of treatment in patients with rheumatic diseases. The HAQ value is used in the management of patients in routine clinical practice. In the following studies: Uutel et al., Garip et al. and Sivas et al., patients with RA demonstrated the effect of daily physical fitness according to the HAQ on the assessment of quality of life among RA patients. These studies showed that the assessment of the quality of life is influenced by the assessment of physical fitness using the HAQ regardless of the occurrence of frailty syndrome [38–40].

The results of our study confirm that the presence of frailty syndrome in RA patients does not affect their quality of life. This may be due to the fact that pain is the main factor that reduces the quality of life of in these patients. Symptoms of RA also often affect the daily lives of patients, their hobbies, earning potential, etc., which in turn translates into a reduction in the quality of life. In the study, Baczyk et al. showed that pain, morning stiffness and grip strength influence the quality of life of RA patients. In our opinion, factors that have been shown by Baczyk significantly reduce the quality of life. Coexistence of frailty syndrome no longer reduces the quality of life assessment by patients. Quality of life assessment is a subjective measurement depending on the patient’s biopsychosocial condition [41].

## Conclusions

Frailty syndrome has no significant impact on the assessment of the quality of life of patients with diagnosed RA. The factors that determine quality of life are different in robust patients and in patients with frailty syndrome. The assessment of the quality of life is affected by the degree of physical fitness regardless of the occurrence of frailty syndrome.

### Limitation of the study

One limitation of this study could be the relatively small study population; however, it was a group of patients that had been diagnosed with RA before they were 60 years of age. Although only one patient answer-based tool was used in the study to assess frailty syndrome, to date, there is no consensus on which tools should be used to assess individual disease entities. In light of the research Salaffi et al., the SHARE-FI tool that was created by Santos-Eggimann et al. may be an even better tool compared to the TFI that we used [27, 28]. Similar situation is with the lately developed tool called CRAF - The Comprehensive Rheumatologic Assessment of Frailty – a multidimensional frailty screening tool in patients with rheumatoid arthritis. Unfortunately, this tool was not available during creation / duration of this research [42].

## Data Availability

The datasets used and analysed during the current study are available from the corresponding author on reasonable request.

## Abbreviations

BMI: Body mass index
CRP: C-reactive protein
DAS28: Disease Activity Score
EL: Energy level
ER: Emotional reaction
ESR: Erythrocyte sedimentation rate
GS: Geriatric syndrome
HAQ: Health Assessment Questionnaire
HRQoL: Health Related Quality of Life
OB: Biernacki reaction
RA: Rheumatoid arthritis
VAS: Visual Analog Scale
